# Biochar from Brewers’ Spent Grain: A Green and Low-Cost Smart Material to Modify Screen-Printed Electrodes

**DOI:** 10.3390/bios9040139

**Published:** 2019-12-03

**Authors:** Rocco Cancelliere, Katya Carbone, Mauro Pagano, Ilaria Cacciotti, Laura Micheli

**Affiliations:** 1 Department of Chemical Sciences and Technologies, University of Rome “Tor Vergata”, Via della Ricerca Scientifica, 00133 Rome, Italy; rocco.cancelliere@uniroma2.it; 2 CREA, Research Centre for Olive, Citrus and Tree Fruit, Via di Fioranello 52, 00134 Rome, Italy; katya.carbone@crea.gov.it; 3 CREA Research Centre for Engineering and Agro-Food Processing, Via Della Pascolare 16, Monterotondo, 00015 Rome, Italy; mauro.pagano@crea.gov.it; 4 Engineering Department, University of Rome “Niccolò Cusano”, Via Don Carlo Gnocchi 3, 00166 Rome, Italy; ilaria.cacciotti@unicusano.it

**Keywords:** brewers’ spent grain, biochar, screen-printed electrode, biosensor, tyrosinase

## Abstract

In the present study, biochar from brewers’ spent grain was used, for the first time, to develop screen-printed electrodes. After having investigated the dispersion behaviour of biochar in different organic solvents, a biochar-based screen-printed electrode was prepared with the drop-casting technique. In order to understand the electrochemical potentiality and performances of the biochar/sensor tool, different electroactive species, i.e., ferricyanide, benzoquinone, epinephrine, ascorbic, and uric acids, were used. The results were compared with those of the same electrodes that were modified with commercial graphene, confirming that the proposed electrode showed improved electrochemical behaviour in terms of resolution, peak-to-peak separation, current intensity, and resistance to charge transfer. Furthermore, a tyrosinase biosensor was developed by direct immobilisation of this enzyme on the biochar/screen printed electrode, as an example of the potential of biochar for disposable biosensor development. The efficiently occurred immobilisation of the biochar on the screen printed electrode’s (SPE’s) surface was demonstrated by the observation of the working electrode with a scanning electron microscope. The detection was performed by measuring the current due to the reduction of the corresponding quinone at low potential, equal to −0.310 V for epinephrine. The experimental conditions for the tyrosinase immobilization and the analytical parameters, such as applied potential and pH of buffer, were studied and optimized. Under these conditions, the electrochemical biosensors were characterized. A linear working range of epinephrine was obtained from 0.05 up to 0.5 mM. The detection limit was 2 × 10^−4^ mM for the biosensor.

## 1. Introduction

The beer brewing process is one of the most polluting industrial processes, generating a huge amount of wastewater effluent and solid wastes (i.e., spent grain and yeast), which must be disposed or treated in the least costly way to meet strict discharge regulations set by government entities [1]. Particularly, spent grain, consisting of grain husks and other residual compounds not converted into fermentable sugars in the mashing process, can constitute as much as 85% of a brewery’s total by-products [2,3]. One of the main challenges of the brewery sector is, therefore, the recovery and valorisation of these wastes through the application of a circular economy model. Brewers’ spent grain (BSG) is available at low or no cost throughout the year and it is produced in large quantities by small and big breweries. Several attempts have been made to recover and valorise BSG in animal feeding; in biotechnological processes, such as cultivation of mushrooms and actinobacteria; and as a source of value-added products, such as phenolic acids or sugar alcohols [4,5]. However, a diffuse employment of BSG as an industrial feedstock is hampered by its chemical deterioration and its susceptibility to microbial attacks due to its high water content (about 70%–80%). The wet and unstable nature of BSG limits the transportation, and the high moisture content prevents efficient or direct energetic utilization. Therefore, an effective treatment of BSG needs to be developed in order to produce a solid, stable carbon source or high-value materials that are beneficial in terms of waste valorisation. In fact, nowadays, the rational use of food waste represents a major challenge in terms of environmental protection, but also from an economic perspective. In this regard, new technologies of pyrolysis and gasification have been developed, especially for thermal processing of biomass [6]. Thermal methods are promising technologies that allow transforming certain types of waste to quality fuel or valuable chemical raw materials. Recently, Sperandio et al. [1] proposed a pyro gasification process for the conversion of BSG into syngas and biochar, which can offer a sustainable approach to by-product disposal to benefit both the environment and craft breweries’ economic outputs. Indeed, this approach allows one to provide energy in terms of syngas for the satisfaction of farms’ energy demands, and at the same time, to replace commercial fertilizer with the use of biochar, obtained from waste biomasses, as a soil improver. Biochar has gained great attention since its production, in combination with its storage in soils, has been suggested as one possible means of reducing the atmospheric CO_2_ concentration. From an agronomic point of view, biochar can improve agricultural productivity, particularly in low-fertility and degraded soils, reducing losses of nutrients, and improve the water-holding capacity of soils [7]. On the other hand, since biochar is a highly porous carbonaceous material, which consists of an inert internal structure and a highly functionalized surface (condensed or residual aliphatic compounds, condensed aromatics) with the ability to interact with different compounds, it is also gaining the attention of analytical chemists [8,9,10].

These structural characteristics of biochar are very similar to those of nanomaterials widely used in electrochemistry (i.e., graphene, nanotubes, and nanofibers), making it a potential alternative for the manufacture of screen printed electrodes (SPEs) based on renewable and biocompatible sources. Nowadays, in fact, there is a growing interest in the use of eco-friendly materials for electronics, giving rise to an innovative generation of high-performance green modifiers [10].

In the literature, several applications of the carbon paste electrode (BCPE), modified with commercial biochar, are reported for the determination of organic and inorganic pollutants in the environment [11,12,13], based on the direct interaction between biochar and pollutants. An example of the application of biochar from tea waste was reported by Bal Altuntas et al. [14] for the development of the glucose biosensor. In that study, the biochar was mixed with graphite and mineral oil to develop the biochar-BCPE and to demonstrate the possibility to use this waste for the production of novel composite electrode. Recently, a review about the disposable electrodes produced by waste materials has been published, where the use of waste paperboard for the support of these electrodes or of vegetables waste for the production of nanoparticles for electrochemical application are reported [10]. Anyway, to the best of our knowledge, no study covering the electroanalytical use of biochar coupled with screen printed electrodes (biochar/SPEs) has been reported in the literature until now. The application of other carbon based materials, such as single-walled carbon nanotubes (SWCNTs), multi-walled carbon nanotubes (MWCNTs) and graphene, has been carried out for the modification of the surface of screen-printed electrodes (SPEs) in order to improve their electrochemical performances. For example, Gomez et al. [15] studied the electrochemical behaviour of SPEs modified with SWCNTs, MWCNTs and graphene, respectively, to detect melatonin (MT) and serotonin (5-HT), with a remarkable improvement in terms of selectivity, reproducibility, and detection limit. Pérez-Ràfols et al. [16] and Apetrei et al. [17], who used SPEs modified with carbon nanofibers, obtained the same improvements in the electrochemical performance. In the present study, biochar from spent grain was used for the first time, in order to modify the graphite-working electrode of SPEs. The modification was carried out by drop casting, using a stable dispersion of biochar (biochar/SPE), demonstrating the possibility of recycling this waste material. At the same time, the SPEs were modified with commercial graphene in order to compare the electrochemical performances (in terms of sensibility, working range, and detection limit) of both electrodes. Moreover, these modified sensors were used as support for the immobilization of tyrosinase, an enzyme selective for catecholamines [18].

In light of these considerations, in the present study, for the first time, biochar from agricultural food waste was used to modify a cheap printed electrode for tyrosinase biosensor development. In this work, the biochar from BSG was studied as an electrochemical enhancer, comparing its performance to those of printed electrodes modified with graphene. This study demonstrated that the use of biochar, instead of graphene, for the fabrication of screen-printed electrode, is very promising, reducing the cost of these devices while increasing the sustainability of beer production.

## 2. Materials and Methods

### 2.1. Chemicals

All reagents were of high purity and they were used without further purification. Graphene, epinephrine (EP), and tyrosinase (Ty) from mushrooms were purchased from Sigma (Merk Life Science S.r.l., Milan, Italy). A 5 mg/mL solution of Ty in 50 mM phosphate buffer (PB), pH 7.4, was used for the enzyme immobilization. The working buffer was 50 mM PB + 10 mM KCl, pH 7.0. Ultrapure water was used for the preparation of all aqueous solutions.

### 2.2. Preparation and Characterization of Biochar From Brewers’ Spent Grain

Pellets from brewers’ spent grain were used as a feedstock for biochar production, as previously reported by Sperandio et al. [1]. Pellets were subjected to a pyrolytic micro-gasification process (T = 400 °C) in an Elsa D17 micro pyrolytic reactor (Bluecomb Ltd., Udine, Italy). After the pyrolysis, biochar samples were manually ground to improve their homogeneity, and then used without further modifications for the sensors’ fabrication [19].

### 2.3. FTIR Analysis and SEM Investigation of Biochar Samples

FTIR spectra of biochar samples were acquired using a Thermo-Scientific instrument (mod. iS 50 Nicolet, Thermo Scientific Inc., Madison, WI, USA), equipped with a single-reflectance horizontal ATR cell with a diamond crystal. Ground and homogenized biochar samples were placed at the surface of the diamond crystal and pressed with a system press tip flap. Samples were scanned at wavenumbers ranging from 4000 to 600 cm^−1^ (scans: 32; scan speed: 0.20 cm/s; resolution: 4 cm^−1^) and corrected against the background spectrum of air. To obtain an averaged spectrum, three replicates of each sample were scanned and averaged. OMNIC™ software (Thermo Fisher Scientific Inc., Waltham, MA, USA) was used for processing the acquired spectra. In order to investigate the morphology of the biochar particles, in terms of shape, average size, and surface porosity, scanning electron microscopy (SEM) micrographs were acquired by means of field emission gun scanning electron microscopy (FEG-SEM) (Cambridge Leo Supra 35, Carl Zeiss, Jena, Germany), after sputter-coating with gold under argon atmosphere (25 mA, 120 s). The average particles diameter and pore sizes were determined considering randomly selected particles from the acquired SEM micrographs (ImageJ, NIH, Bethesda, MA, USA).

### 2.4. Electrochemical Characterization of Biochar Samples

All the electrochemical characterizations were performed by cyclic voltammetry (CV) and amperometry (CA), using screen printed electrodes (SPEs), home produced by the Laboratory of Analytical Chemistry of the University of Rome “Tor Vergata.” The diameter of the working electrode was 0.3 cm, resulting in a geometric area of 0.07 cm^2^. The measurements were carried out using an Autolab electrochemical system (Eco Chemie, Utrecht, The Netherlands), equipped with PGSTAT-12 and GPES software (Eco Chemie, Utrecht, The Netherlands)) [20,21].

### 2.5. Biochar Modified SPEs (Biochar/SPE) Preparation and Characterization

The biochar dispersions were prepared in ethanolic medium at a concentration of 1 mg/mL, and then treated in an ultrasonic bath for 60 min. The biochar/SPEs were assembled via drop casting with 6 μL of biochar dispersions on bare graphitic SPE; then, the solvent was allowed to volatilize (37 °C). Electrochemical measurements were performed in drop (70 µL) detection mode. To evaluate the biochar distribution on the SPE, biochar/SPE was observed at FEG-SEM, after sputter-coating with gold under argon atmosphere (25 mA, 120 s), and compared with bare SPE.

### 2.6. Graphene-Modified SPEs (Graphene/SPE)

Graphene dispersions were prepared following the procedure described by Cinti et al. [21]: 10 mg of commercial reduced graphene oxide powder (rGO) was added to 10 mL of solvent (a mixture dimethylformamide (DMF): water (1:1, v/v)) and sonicated for 60 min at 59 kHz. Using this solution, the SPEs were modified via drop casting, adding 6 µL of graphene dispersion (1 mg/mL) on a working electrode, as described in detail in Section 2.5.

### 2.7. Fabrication and Characterization of Biosensor

The biosensor (Ty/biochar/SPE) was prepared immobilizing Ty on biochar/SPE by drop casting technique, followed by cross-linking with glutaraldehyde. A quantity of 50 µL of 50 mM PB containing 5 mg/mL of Ty was added onto the graphite biochar modified working electrode. After drying, the Ty/biochar/SPE was exposed to a 2.5% (v/v) glutaraldehyde solution (in 50 mM PB + 10 mM KCl, pH 7.0) for 20 min at room temperature. The enzyme-immobilized film was dried at room temperature and rinsed with PB to remove any unbound enzyme from the electrode surface. Finally, the biosensors were stored at 4 °C.

To study the repeatability, the stability, and the storage of the Ty/biochar/SPE biosensor, amperometric measurements were carried out with different EP solutions, in 50 mM PB at pH 7.0, using the same biosensor device. To demonstrate that enzymatic immobilization had occurred on the modified biochar/SPE, SEM micrographs were acquired by means of FEG-SEM, after sputter-coating with gold under argon atmosphere (25 mA, 120 s).

## 3. Results and Discussions

### 3.1. FTIR Analysis of Biochar Samples

**Figure 1:**
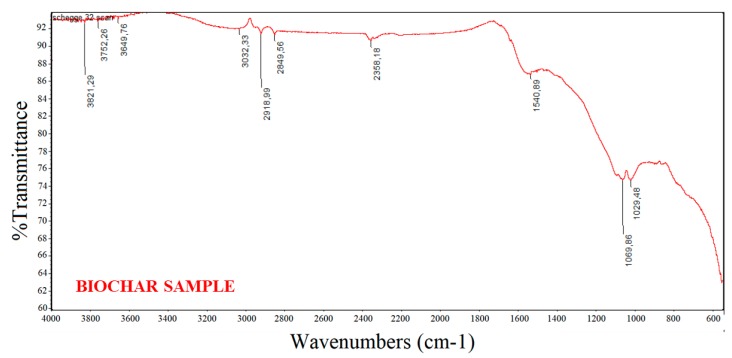
FTIR spectrum of the biochar sample.

In the present study, FTIR spectroscopy was used to analyse the functional groups on the surface of the biochar particles employed for sensor fabrication. In fact, besides the porosity, the adsorption behaviour of a biochar is influenced by the chemical reactivity of its surface, especially in the form of chemisorbed oxygen in various functional groups. Figure 1 shows a typical, averaged FT-IR spectrum (media of 16 spectra) of the biochar material investigated. The absence of the bands due to aromatic C–H stretch at 3050 cm^−1^ and aliphatic C–H stretch at 2900 cm^−1^ suggests that BSG biochar is comprised of two main structural fractions: graphite-like and randomly ordered, amorphous aromatic structures [22]. The IR spectrum of BSG biochar was characterized essentially by a restricted group of frequencies linked to the presence of aryl ring and phenol features. The peaks at around 2360 and 2340 cm^−1^ indicate carboxyl and carbonyl groups. The aromatic ring vibrations in the wavenumber range around 1600–1450 cm^−1^ confirm the presence of the aforementioned aromatic structures (C=C–C absorption bands) [23,24]. The peaks between 1350 and 1050 cm^−1^ can be ascribed to the presence of primary, secondary, and tertiary alcohols; phenols; ethers; and esters showing C–O stretching and O–H deformation vibrations.

Finally, the peaks between 850 and 630 cm^−1^ correspond to aromatic C–H stretching vibrations that indicate the presence of adjacent aromatic hydrogens in biochar samples [25].

**Figure 2:**
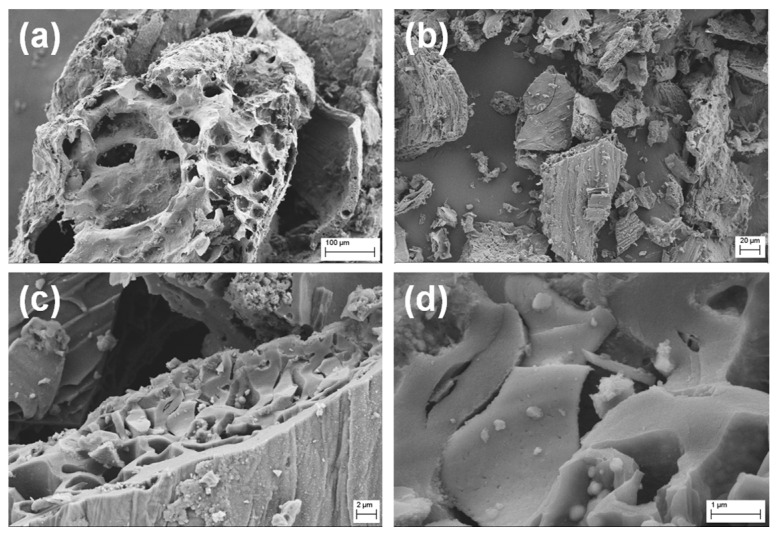
SEM micrographs of the biochar sample at different magnifications: (**a**) 500×, (**b**) 1000×, (**c**) 10,000×, (**d**) 50,000×.

The morphologies of the biochar samples were analysed at SEM. Figure 2a–d shows low and high magnification SEM micrographs of the biochar from BSG.

Figure 2b underlines that biochar powder was composed of irregularly shaped, significantly amorphous and heterogeneous macro- and micro-particles with sponge-like structures, characterized by the presence of several pores of different sizes and many hollow channels (average diameters around 10–20 µm), even if little defined, uneven, and not uniform (Figure 2a). It is possible to observe very coarse, heterogeneous and plane cleavage surfaces, due to the pyrolysis process that is able to stabilize the volatile hydrocarbons, smoothening the biochar surface, and broken edges with tarry deposits on the surface. The vesicles on the biochar surface resulted from the gradual release of different volatile compounds formed in the softened biomass matrix during the pyrolysis process through a melt phase of cellular components (Figure 2c,d) [25,26,27,28].

It is important to take into account that the lignin and high volatile matter content in the starting biomass waste significantly affects the formation of porosities in the resulting biochar sample [29]. Moreover, the heat transfer during the gasification process strongly depends on the bulk density of the starting biomass waste material because lower ratios of air/fuel are achieved for lower bulk densities (i.e., lesser amount of biomass in the same volume of reactor) [30]. Generally, the number of pores and their size increase with the pyrolysis temperature (up to 550 °C), with consequent increment of the specific surface area as a function of temperature [31]. However, the process tends to combustion, increasing both temperature and reaction velocity, and higher carbonization degrees correspond to an increase in the carbon amount and a decrease in the oxygen content of the biochar, with a resultant increment of its surface hydrophobicity [30]. For these reasons, the pyrolysis was performed at 400 °C. Indeed, it has been reported that at pyrolysis temperatures higher than 550 °C, the biochar has a lower specific surface area due to the shrinkage of chars at post-softening and swelling temperatures, resulting in narrowing or closing pores, and a distortion of the pore structures occurs starting from 700 °C [32]. Indeed, it is very important to obtain and preserve these porous structures, since they provide a high internal surface area, and thus, high adsorption ability.

### 3.2. Biochar-Modified SPEs (Biochar/SPE)

With the aim of expanding its processability and future practical applications for SPE modification, we previously investigated the dispersion behaviour of biochar in different organic solvents. In this study, three different solvents were used for the dispersion of biochar: *N*,*N*-dimethylformamide (DMF)/H_2_O (1:1, v/v), 1-Methyl-2-pyrrolidinone (NMP), and ethanol.

**Figure 3:**
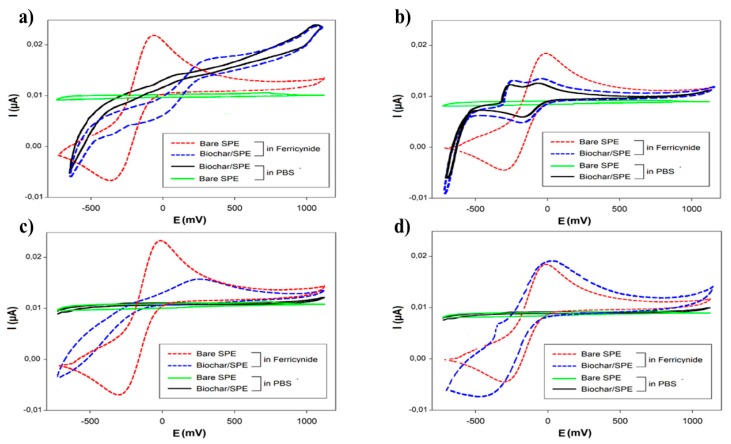
Cyclic voltammograms of bare screen printed electrodes (SPEs) and biochar/SPEs sensors obtained with dispersion of (**a**) DMF:H_2_O (1:1 v/v), (**b**) DMF, (**c**) NMP, or (**d**) ethanol. Measurement conditions: scan rate 30 mVs^−1^.

These dispersions (1 mg/mL), after sonication (60 min, 59 kHz), were used to modify the surface of the working electrode (WE) via drop casting; then the electrochemical response was tested and compared using cyclic voltammetry (CV) (Figure 3). Two test solutions were used: 50 mM PB + 10 mM KCl, pH 7.0, and 1 mM ferricyanide (K_3_Fe(CN)_6_), respectively (70 µL/SPE).

The results, shown in Figure 3, indicated that the solvent to be used for the modification of SPEs had to be the ethanol, because it guaranteed the most homogenous dispersion of the biochar, in relation to the recorded electrochemical signal. Using this solvent, the voltammogram of ferricyanide was closer to the ideal reversible behaviour of this compound; for this reason, it was chosen as the working solvent.

**Figure 4:**
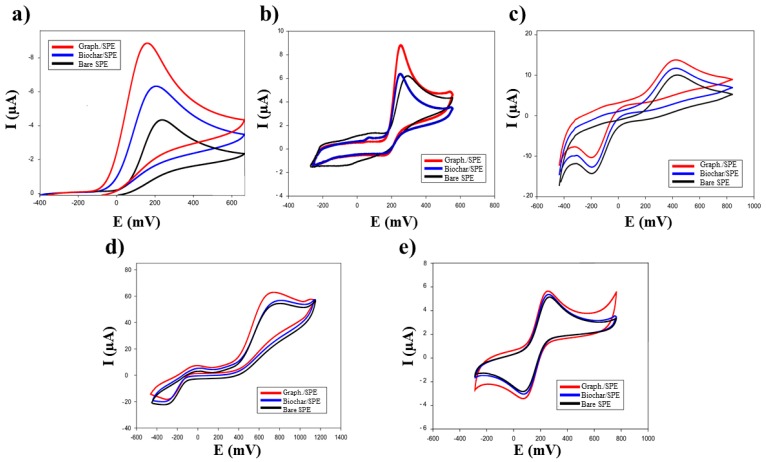
Cyclic Voltammograms of biochar based, graphene based, and bare SPE sensors obtained with: (**a**) 20 mM ascorbic acid, (**b**) 20 mM uric acid, (**c**) 20 mM benzoquinone, (**d**) 20 mM epinephrine, and (**e**) 20 mM ferricyanide in 50 mM phosphate buffer + 10 mM KCl, pH 7.4; scan rate 30 mVs^−1^.

The electrochemical characterization of biochar/SPEs (*n* = 6) was carried out using five different electroactive compounds (Figure 4, Table 1): ferricyanide, epinephrine (EP), benzoquinone, uric acid (UA), and ascorbic acid (AA).

The results obtained with biochar/SPEs were compared with those of commercial graphene/SPE sensors, using the same substrates (Table 1).

The performances of biochar/sensors were comparable to those based on commercial graphene, widely used for this type of modification [17]. This experimental evidence shows that biochar can actually be used as an electrochemical enhancer with electrochemical performance similar to that of commercial graphene.

### 3.3. Electrochemical Behavior of Biochar/SPE

**Figure 5:**
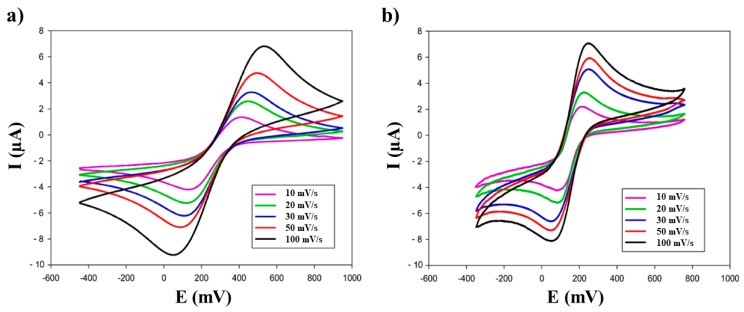
Scan rate study: comparison of cyclic voltammograms obtained with (**a**) biochar/SPEs and (**b**) graphene/SPEs sensors using 10 mM ferricyanide in 50 mM phosphate buffer + 10 mM KCl, pH 7.4.

A further investigation was carried out in order to understand the electrochemical behaviour of the biochar/SPE interface (Figure 5, Table 2).

The voltammetric peak heights (I_p_), obtained for biochar and graphene modified-SPEs in the scan rate study (Figure 5), were plotted against the square root of the scan rate. The following slopes (µAcm^−1^s^−1/2^) were obtained: 0.86 (R^2^ = 0.997), 0.82 (R^2^ = 0.995), and 0.47 (R^2^ = 0.992), corresponding to graphene/SPE, biochar/SPE, and bare SPE, respectively.

The effective electrode area was calculated (Table 2) by Randless–Sevcik equation (Equation (1)) [33,34] using the diffusion coefficient *D*_0_ and *D_R_*, described by Konopka et al. (i.e., *D*_0_ = 7.26 × 10^−6^ cm^2^/s, *D_R_* = 6.67 × 10^6^ cm^2^s^−1^), and the *I_p_* values, obtained for scan rate 30 mVs^−1^:

(1) $$I_{p}=\left(0.4463\right)nFAC\sqrt{\frac{nF\nu D_{0}}{RT}},$$

where *F* is the constant of Faraday (mol^−1^), *R* the universal constant of gas (JK^−1^mol^−1^), *n* the number of electrons exchanged, *A* the electrodic surface (cm^2^), *C* the analyte concentration (molcm^−3^), *D*_0_ the diffusion coefficient (cm^2^s^−1^), and *ν* the scan rate (mVs^−1^), respectively.

Furthermore, the heterogeneous rate constants (*k*^0^) for the redox process [Fe(CN)_6_]^3−^ + 1e^−^ ⇆ [Fe(CN)_6_]^4−^ were calculated, using the Equation (2):

(2) k0=[D0πν(nFRT)]12(D0DR)α2φ

where *D*_0_ and *D_R_* are the diffusion coefficients for the ferricyanide (*D*_0_) and ferrocyanide (*D_R_*), *ν* is the scan rate (Vs^−1^), *n* is the number of electrons involved in the process, *F* is the Faraday constant (mol^−1^), *T* is the temperature (K), *R* is the universal gas constant (JK^−1^mol^−1^), and *α* the dimensional transfer coefficient [34].

For the *D*_0_ and *D_R_*, the relative values described by Konopka et al. (i.e., *D*_0_ = 7.26 × 10^−6^ cm^2^/s, *D_R_* = 6.67 × 10^6^ cm^2^s^−1^) were used [34]. The α parameter was chosen to be equal to 0.5, assuming the ratio of the anodic and cathodic peak equal approximately to 1 (*I*_pa_/I_pc_ = 1).

The parameter *ϕ* can be obtained using the Nickolson method [35], where for each Δ*E* there is a correspondence with a Ψ value. For a better evaluation of this parameter, the equation based on the Nickolson theory was used [36]:

(3) $$\Psi =\frac{\left(-0.6288+0.0021\cdot \Delta E\right)}{\left(1-0.017\cdot \Delta E\right)}$$

The results obtained show a variation in the heterogeneous rate constants, demonstrating that biochar modified SPEs have a slower process of electronic transfer than the graphene modified platforms (Table 3).

### 3.4. Tyrosinase Biosensor: An Example of Biochar/SPE Application

Liu at al. [37] reported an interesting work about the development of a tyrosinase/Chitosan/GOx SPE with a good sensitivity (22 nM) and a broad linear range (0.1–500 µM) compared with existing electrochemical sensors. Taking inspiration from the literature, biochar was used, for the first time, for the fabrication of biosensors by using biochar/SPE as the support for tyrosinase (Ty) immobilization, in order to demonstrate the possibility of using it like the widely commercial nanomaterials. This Ty/Biochar/SPE was designed for the development of biosensors with an enhanced electrochemical active area and enhanced electronic transfer properties. The enzymatic substrate chosen was epinephrine (one of the catecholamines).

**Figure 6:**
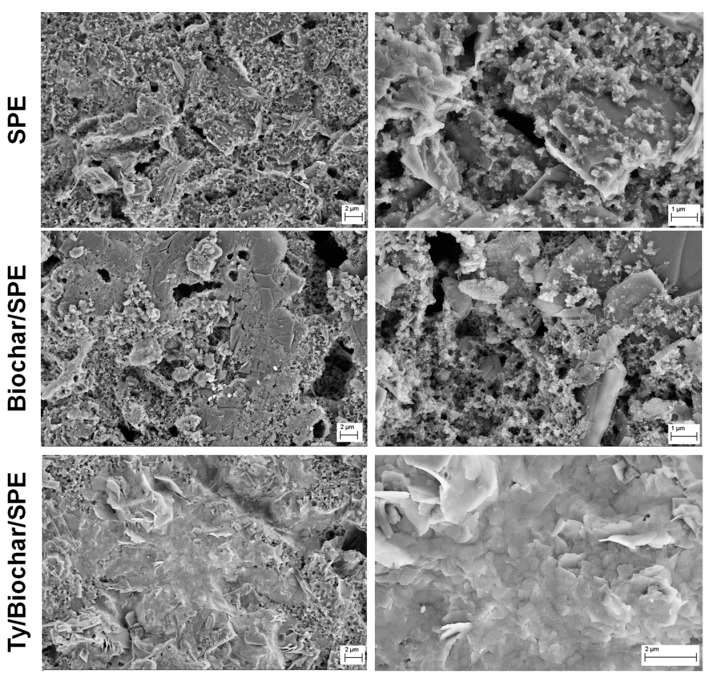
SEM micrographs of bare SPE, biochar/SPE and Ty/biochar/SPE.

In order to investigate the enzyme immobilization on the surface of the biochar modified SPEs, the bare SPE, biochar/SPE, and Ty/biochar/SPE samples were observed by means of SEM. Figure 6 compares their low and high magnification SEM micrographs. In all cases, low magnification micrographs allowed us to demonstrate the uniform and homogeneous distribution of the used ink on the surface of the working electrode, characterized by graphite micrometre-sized flakes bound together with an inert polymeric binder and covered of small cross-linking particles, present in the ink we employed. Additionally, biochar/SPE presented structures ascribable to the biochar addition, even if smaller than the starting particles, due to the ultrasonic treatment applied in order to obtain a good dispersion in the ethanol. In the case of Ty/biochar/SPE, the presence of a uniform and homogeneous layer on the biochar/SPE is evident, testifying that the enzyme immobilization allows for a homogenous and well-anchored enzymatic membrane.

**Figure 7:**
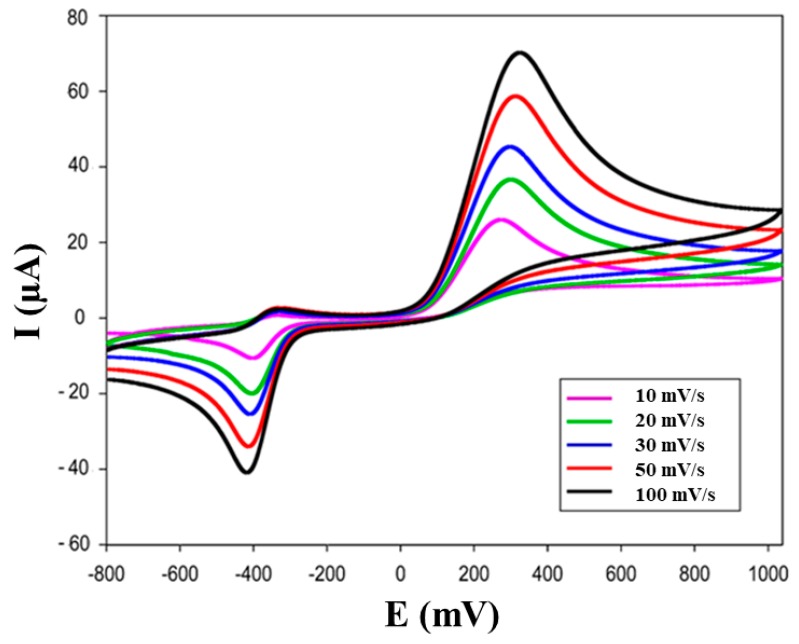
Cyclic Voltammograms of Ty-biochar/SPE biosensor of scan rate study: comparison of cyclic voltammograms obtained with Ty/biochar/SPEs biosensors using 2 mM epinephrine solution (in 50 mM phosphate buffer + 10 mM KCl, pH 7.4).

The cyclic voltammograms are shown in Figure 7 using 100 μM epinephrine in 50 mM PB + 10 mM KCl, pH 7.0, where a reversible electrochemical behaviour was observed for epinephrine.

The oxidation peak is due to the oxidation of catecholamine to o-quinone, as shown in the following Reaction (1) [17,18]:

Epinephrine + Tyrosinase (O_2_) → o-Epinephrinequinone + H_2_O. (1)

The o-epinephrinquinone is electrochemically reduced to epinephrine on biosensor surface (2):

o-Epinephrinequinone + 2H^+^ + 2e^−^ → Epinephrine. (2)

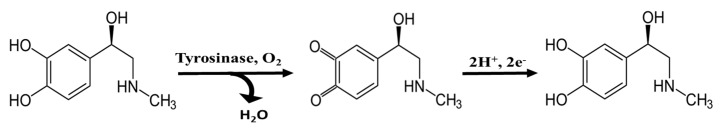


In EP solution (supporting electrolyte 50 mM PB, pH 7.0), the CV gave two well-defined peaks: cathodic peak at −0.030 V and anodic peak at +0.25 V.

These experimental data demonstrate that the tyrosinase enzyme retains its bioactivity when immobilized on biochar thick film. Tyrosinase, immobilized on biochar/SPE, efficiently catalyses the oxidation of EP. The sharp and intense oxidation and reduction peaks reveal a fast electron transfer at tyrosinase immobilized on biochar/SPE [17,18]. These results confirm that this method allows the determination of compounds belonging to the catecholamine family with satisfactory results, as also explained by Arduini et al. [38] and Maciejewska et al. [39].

Additionally, the effect of potential scan rate on the current peak of this catecholamine was studied.

In Figure 7, it can also be seen that the oxidation peak shifts to a more positive value, while the reduction peak to more negative values proportional to the increasing scan rates with the increment of the current for this catecholamine.

BSG biochar presents, not only, similarities with graphene, such as the ability to improve electrochemical performance and to be easily dispersed in stable and homogeneous suspensions, but has benefits such as cost-effectiveness—being configured as a cheap and easy-to-use material—for the development of electrochemical sensors.

The characterization of biochar as a supporting material to modify the SPE was carried out studying several parameters, such as the pH of the working buffer used for the electrochemical measurements, working range, stability, and reproducibility of the biosensor, and the storage conditions of the biochar-based biosensor. The influence of the pH (in the range of 4.0–9.0) on the electrochemical behaviour of EP in several buffer solutions was investigated using cyclic voltammetry. The pH of working buffer showed a significant effect on the electrochemical behaviour of EP (100 μM) at the surface of the modified biosensor.

**Figure 8:**
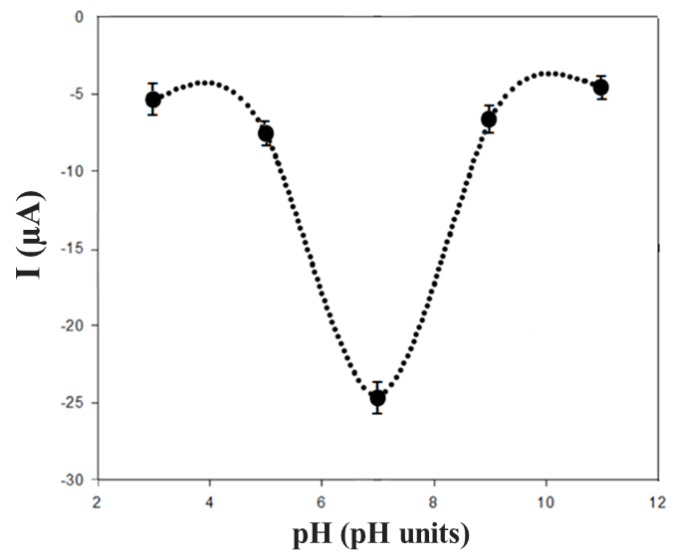
Current-pH dependence in 10 mM EP solution (in 50 mM phosphate buffer + 10 mM KCl, at different pH values).

It was observed that the peak potentials decreased (with a shift towards negative potential values) with the increase of the pH. In Figure 8, the graph of the cathodic peak current (Ipc) versus pH is reported. The better electrochemical performances were obtained when the pH was 7.0.

**Figure 9:**
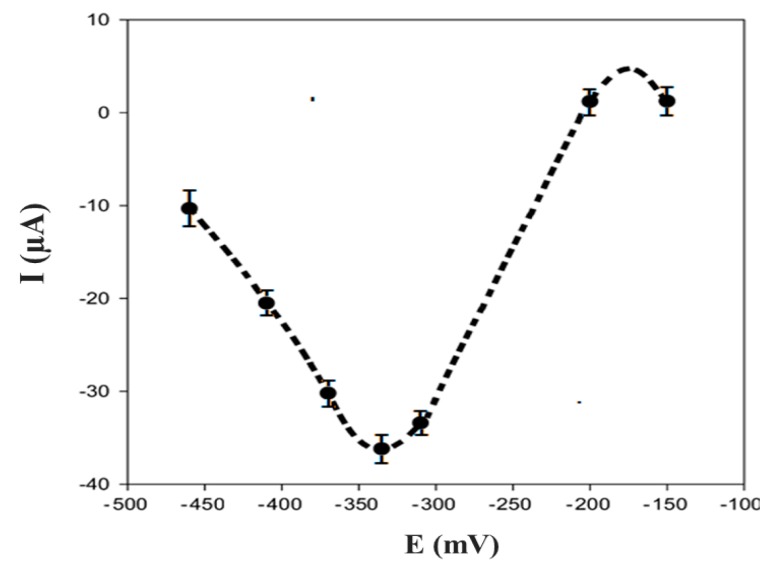
Current-potential dependence in 10 mM EP solution (in 50 mM phosphate buffer + 10 mM KCl, pH 7.0).

The dependence of the biosensor’s response on the electrode potential is shown in Figure 9 using 100 μM EP in 50 mM PB + 10 mM KCl, pH 7.0. The maximum of the electrochemical signal was obtained at −0.32 V; this potential was applied to EP detection after the immobilization of a specific enzyme for this molecule.

### 3.5. Amperometric Response of the Biosensor

**Figure 10:**
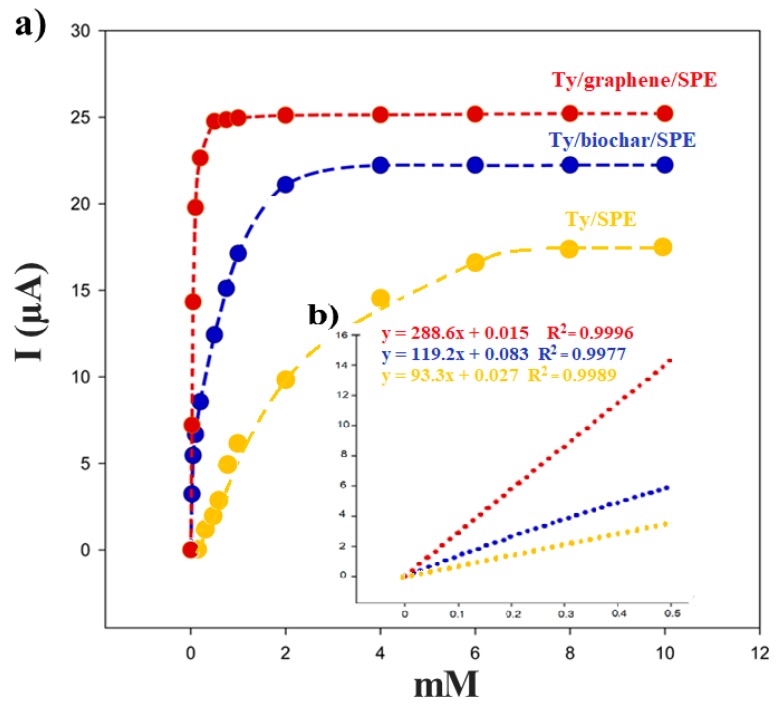
(**a**) Calibration curve between the cathodic current and the concentration of EP in 50 mM phosphate buffer + 10 mM KCl, pH 7.4: (blue) Ty/biochar/SPE, (red) Ty/GPH/SPE, and (yellow) Ty/bare SPE biosensor. (**b**) Linear range.

Figure 10 illustrates a typical amperometric response for the Ty/biochar/SPE biosensor after the addition of successive aliquots of EP using 50 mM PB + 10 mM KCl (pH 7.0) and applying −0.32 V as the working potential. The reduction of current, proportional to the concentration of catecholamine, was due to the electrochemical reduction of o-epinephrinequinone, the enzymatic product of tyrosine when EP is present.

The measurements of EP showed a linear current response in a concentration range of 0.05–0.5 mM (Figure 10). The detection limit, calculated as *3sb/m* criterion (*m* is the slope of the calibration graph; *sd* is the standard deviation (*n* = 5) of the current signals of the substrate at the concentration level corresponding to the lowest concentration of the calibration plot) [40]) resulted in 2.4 × 10^−4^ mM. This value was within the range of the detection limit values found for tyrosinase biosensors present in literature [18].

Using these results, the Hill coefficient (h) can be calculated as the log[*I*/(*I_max_* − *I*)] versus log [EP] (the logarithm of the substrate concentration), obtaining 0.89 for biochar/SPE biosensor (r^2^ = 0.990) and 1.01 for graphene/SPE biosensor (r^2^ = 0.999), respectively. The h parameter, calculated from the corresponding Hill’s plot, was close to 1, demonstrated that the kinetics of the enzymatic reaction fitted into a Michaelis–Menten kinetics model [41]. The enzymatic kinetic parameters were obtained using the linearization of Lineweaver–Burk by Equation (4) [40]:

(4) $$\frac{1}{I}=\frac{1}{I_{max}}+\frac{K_{M}^{app}}{I_{max}\left[S\right]},$$

where [*S*] is the concentration of the substrate, *I* the cathodic current at −0.32V, and $K_{M}^{app}$ the apparent Michaelis–Menten constant for the enzymatic reaction and *I_max_* the steady-state current. Using the Lineweaver–Burk equation and representing 1/*I* versus 1/[EP], the apparent Michaelis-Menten constant was calculated from the slope and the *I_max_* from the intercept.

From the data shown in the Table 4 we can see a greater affinity of the enzyme when it is immobilized on graphene; however, as for all the experimental results obtained in this work, in this case, the biosensor performance based on the biochar is comparable with that of Ty/graphene/SPE.

### 3.6. Repeatability and Stability of the Biosensor

To study the repeatability of Ty/biochar and graphene based SPEs, amperometric measurements for epinephrine detection were carried out with a 0.25 mM EP solution in PB, using the same biosensors devices for 10 times. The relative standard deviation (%RSD) for the Ty/biochar/SPEs was equal to 6%, while for Ty/graphene platform it was 4%.

The stability of the biosensor was studied by monitoring the amperometric response of the same biosensor at regular intervals of 24 hours for 20 days. Between measurements, the biosensor was stored in a refrigerator at 4 °C in dry condition. The amperometric responses of the biosensor with and without biochar remained quite constant in the first 7 days of storage. In particular, biochar-based biosensor maintained 98.2% of its initial current response with a decrease to 87.6% after two weeks, while the Ty/graphene-SPE showed an initial 98.5%, decreasing to 92.2% after two weeks. After this time, the biosensor’s amperometric response constantly decreased, until 55.7% (biochar) and 75.6% (graphene) of the initial signal in 20 days. Therefore, both graphene and biochar-based platforms can be considered “ready to use” and stored at 4 °C within the first 7–10 days, obtaining good results for EP determination.

## 4. Conclusions

In the present study, brewers’ spent grain biochar was investigated as an innovative, eco-friendly graphene-alternative for the production of screen-printed electrodes.

From SEM investigation, it is evident that the biochar powder we produced was made of irregularly shaped macro- and micro-particles with a sponge-like structure, characterized by the presence of several pores of different sizes and many hollow channels, suitable for enzymes’ immobilization and electrochemical applications. Following this approach, the biochar obtained from spent grain was used to improve the performances of screen-printed electrodes through the modification of their surfaces with the proposed material by drop casting. Moreover, the good and uniform distribution of biochar on the SPE surface was demonstrated by observation at SEM, as well as the tyrosinase immobilization in the case of Ty/biochar/SPE. The Ty/biochar/SPEs exhibited similar characteristics to graphene/SPEs, widely used for biosensor development, in terms of the electron transfer kinetics for electroactive compounds. Large peak potential separation and high peak current could be obtained using CV on the developed electrode. Good sensitivity and detection limit for epinephrine promote the Ty/biochar/SPE to be an effective sensor for direct determination of this molecular target in real samples.

## Figures and Tables

**Table 1:** Comparison of anodic peak current repeatability for biochar/SPE and graphene/SPE sensors for different electrochemical substrates (RSD% = standard deviation/µA, *n* = 5).

	Biochar/SPE RSD%	Graphene/SPE RSD%
Ascorbic acid	13	2
Uric acid	13	6
Benzoquinone	12	2
Epinephrine	12	1
Ferrcyanide	12	4

**Table 2:** Comparison of the effective active area for two different biochar/SPE and graphene/SPE sensors.

	A (cm^2^)		M ± σ
	Anodic	Cathodic	
biochar/SPE	0.041	0.045	(4.3 ± 0.5) × 10^−2^
graphene/SPE	0.053	0.049	(5.1 ± 0.6) × 10^−2^

**Table 3:** Comparison of *k*^0^ standard electron rate constant for biochar/SPE and graphene/SPE sensors.

	*k*^0^ (cms^−1^)
biochar/SPE	(2.3 ± 0.1) × 10^−3^
graphene/SPE	(1.6 ± 0.1) × 10^−3^

**Table 4:** Comparison of analytical parameters for different biosensors: Ty/bare SPE, Ty/Biochar/SPE, and Ty/Graphene/SPE biosensors.

	Linear Range (mM)	LOD (mM)	$K_{M}^{app}$ (mM)
Ty/biochar/SPE	0.02–0.50	2.4 × 10^−4^	0.15
Ty/graphene/SPE	0.02–0.25	1.0 × 10^−4^	0.09
Ty/bare SPE	0.02–2.0	9.2 × 10^−4^	0.25
